# Effects of Age, Exercise Duration, and Test Conditions on Heart Rate Variability in Young Endurance Horses

**DOI:** 10.3389/fphys.2016.00155

**Published:** 2016-05-02

**Authors:** Mohamed Younes, Céline Robert, Eric Barrey, François Cottin

**Affiliations:** ^1^CIAMS, Université Paris-Sud, Université Paris-SaclayOrsay, France; ^2^CIAMS, Université d'OrléansLoiret Orléans, France; ^3^GABI, Institut National de la Recherche Agronomique, AgroParisTech, Université Paris-SaclayJouy-en-Josas, France; ^4^Ecole Nationale Vétérinaire d'Alfort, Université Paris-EstMaisons-Alfort, France

**Keywords:** performance, rest, exercise, cardiac recovery, speed, training

## Abstract

Although cardiac recovery is an important criterion for ranking horses in endurance competitions, heart rate variability (HRV) has hardly ever been studied in the context of this equestrian discipline. In the present study, we sought to determine whether HRV is affected by parameters such as age, exercise duration and test site. Accordingly, HRV might be used to select endurance horses with the fastest cardiac recovery. The main objective of the present study was to determine the effects of age, exercise duration, and test site on HRV variables at rest and during exercise and recovery in young Arabian endurance horses. Over a 3-year period, 77 young Arabian horses aged 4–6 years performed one or more exercise tests (consisting of a warm-up, cantering at 22 km.h^−1^and a final 500 m gallop at full speed) at four different sites. Beat-to-beat RR intervals were continuously recorded and then analyzed (using a time-frequency approach) to determine the instantaneous HRV components before, during and after the test. At rest, the root-mean-square of successive differences in RR intervals (RMSSD) was higher in the 4-year-olds (54.4 ± 14.5 ms) than in the 5-or 6-year-olds (44.9 ± 15.5 and 49.1 ± 11.7 ms, respectively). During the first 15 min of exercise (period T), the heart rate (HR) and RMSSD decreased with age. In 6-year-olds, RMSSD decreased as the exercise duration increased (T: 3.0 ± 1.4 vs. 2T: 3.6 ± 2.2 vs. 3T: 2.8 ± 1.0). During recovery, RMSSD was negatively correlated with the cardiac recovery time (CRT) and the recovery heart rate (RHR; R = −0.56 and −0.53, respectively; *p* < 0.05). At rest and during exercise and recovery, RMSSD and several HRV variables differed significantly as a function of the test conditions. HRV in endurance horses appears to be strongly influenced by age and environmental factors (such as ambient temperature, ambient humidity, and track quality). Nevertheless, RMSSD can be used to select endurance horses with the fastest cardiac recovery.

## Introduction

Equestrian endurance competitions (ranging in distance from 80 to 160 km) are split into successive phases of ~30–40 km. At the end of each phase, horses are checked in a veterinary inspection (referred to as a “vet gate”). The heart rate (HR) is the primary criterion evaluated at the vet gate. The horse will fail the vet gate inspection (and will thus be disqualified) if its heart rate is still above 64 bpm after 20 min of recovery (FEI Endurance rules, 2016: p. 31–32). Consequently, rapid cardiac recovery is a key criterion for success in endurance events and is considered to be a reliable indicator of fatigue during these races (FEI, 2016).

Regarding physiological aspects, HR recovery is controlled by the balance between parasympathetic and sympathetic efferent controls (the sympathovagal balance). Heart rate variability (HRV) analysis is a useful tool for quantifying cardiac autonomic control at rest (Filliau et al., 2014) and during exercise in humans (Cottin et al., 2007, 2008) and other mammals (including horses) (Kuwahara et al., 1999). In both horses (Kuwahara et al., 1999; Couroucé et al., 2002) and humans (Carter et al., 2003a,b), it has been shown that long-duration training is associated with (i) a decrease in HR during exercise and recovery and (ii) an increase in HRV at a given running speed. However, cardiac activity and HRV are influenced by several parameters, such as age, exercise duration, and environmental conditions.

In humans, HRV is strongly influenced by age: it has been shown that the HR falls progressively from childhood to adolescence, whereas the amplitude (the square root of the instantaneous power) of HRV increases (Silvetti et al., 2001). In adults, a decrease in the parasympathetic modulation of HR entails a decrease in HRV amplitude with age (Carter et al., 2003a; Antelmi et al., 2004). However, exercise training slows this age-related decrease in HRV (Tulppo et al., 1998; Carter et al., 2003a). To the best of our knowledge, HRV has not previously been studied in endurance horses, and there are few literature data on the effect of a horse's age on physiological parameters. For instance, Seeherman and Morris (1991) showed that the maximum HR (HR_max_) did not change with age in yearling vs. adult Thoroughbred horses, whereas aerobic power and exercise capacity increased with age and training. When considering cardiac autonomic control and given that left ventricular volume and stroke volume are greater in 6-year-old endurance horses than in 4-year-olds (Trachsel et al., 2014) for the same cardiac output, HR should be lower in 6-year-olds than in 4-year-olds. These changes probably mean that HRV differs in 4- vs. 6-year-olds.

In contrast, the effect of exercise duration on HRV components in horses is better documented. A study of elite trotting horses (Cottin et al., 2006) showed that HRV decreased during high intensity interval training. Furthermore, Amory et al. (2010) reported that left ventricular systolic function decreased in horses competing in long-duration races. Accordingly, we hypothesized that HRV components decrease in endurance horses as the exercise duration increases.

Performance in endurance competitions is also affected by external conditions, such as weather conditions (Marlin et al., 1995; Nagy et al., 2014) and the type of track surface (Setterbo et al., 2009). A study by Marlin et al. (2001) showed that in hot, humid conditions, horses breathe more rapidly and have a higher heart rate than they do in cold conditions. Hence, we hypothesized that HRV vagal components decrease under hot and humid conditions.

Current methods for selecting the best endurance horses are based solely on performance indices (such as running speed and race ranking). However, in humans, physiological parameters (VO_2_max and cardiac parameters) are additionally used to select endurance athletes. Accordingly, we hypothesized that an analysis of HRV in young endurance horses (aged 4–6 years) could be used to detect those with the greatest athletic potential (i.e., the fastest cardiac recovery at the vet gate). Hence, with a view to selecting the best young endurance horses, the objective of the present study was to identify HRV components related to fast cardiac recovery. To this end, we determined the effects of age, exercise duration and test site on HRV components at rest, during exercise and during recovery in young endurance horses.

## Methods

### Horses

Between 2012 and 2014, 96 Arabian and Arabian cross endurance horses participated in the study. The horses were aged between 4 and 6 and came from across France. Nineteen of the tested horses were excluded definitively from the final analysis, due to either poor-quality cardiac recordings (*n* = 18) or failure to complete the test (lameness, *n* = 1). For the remaining 77 horses, we excluded six poor-quality cardiac recordings at rest, 23 poor-quality recordings made during the first 15 min of exercise, and 16 poor-quality recordings made during the recovery period.

The characteristics of the remaining 77 horses are presented in Table 1 by age and by test site. Information on each horse's training and racing profile (such as the number of races, the total distance covered during races and the weekly duration of training) was obtained from the French Equestrian Federation's database (*FFE compet*) or a questionnaire completed by the owner.

**Table 1:** **Distribution of the participating horses, by age and test site**.

***N* total = 77**	**Test site**			
Age (period)	A (2012–2013)	C2 (2014)	L (2012–2014)	C1 (2012–2014)
4 years (*n* = 23)	8	1	7	7
5 years (*n* = 34)	7	5	12	10
6 years (*n* = 20)	5	2	6	7
Average ambient temperature and humidity	27°C/42%	26°C/60%	18°C/73%	20°C/55%
Track surface	Hard field	Hard field	Soft sand	Soft sand

The study was approved by the local institutional ethics committee (ComEthANSES/ENVA/UPEC, Maisons-Alfort, France; approval number: 12/07/11-1) and meet this journal's ethical standards. All horse owners provided their written, informed consent and were told that they could withdraw their horse from the study at any time.

### Experimental design

**Figure 1:**
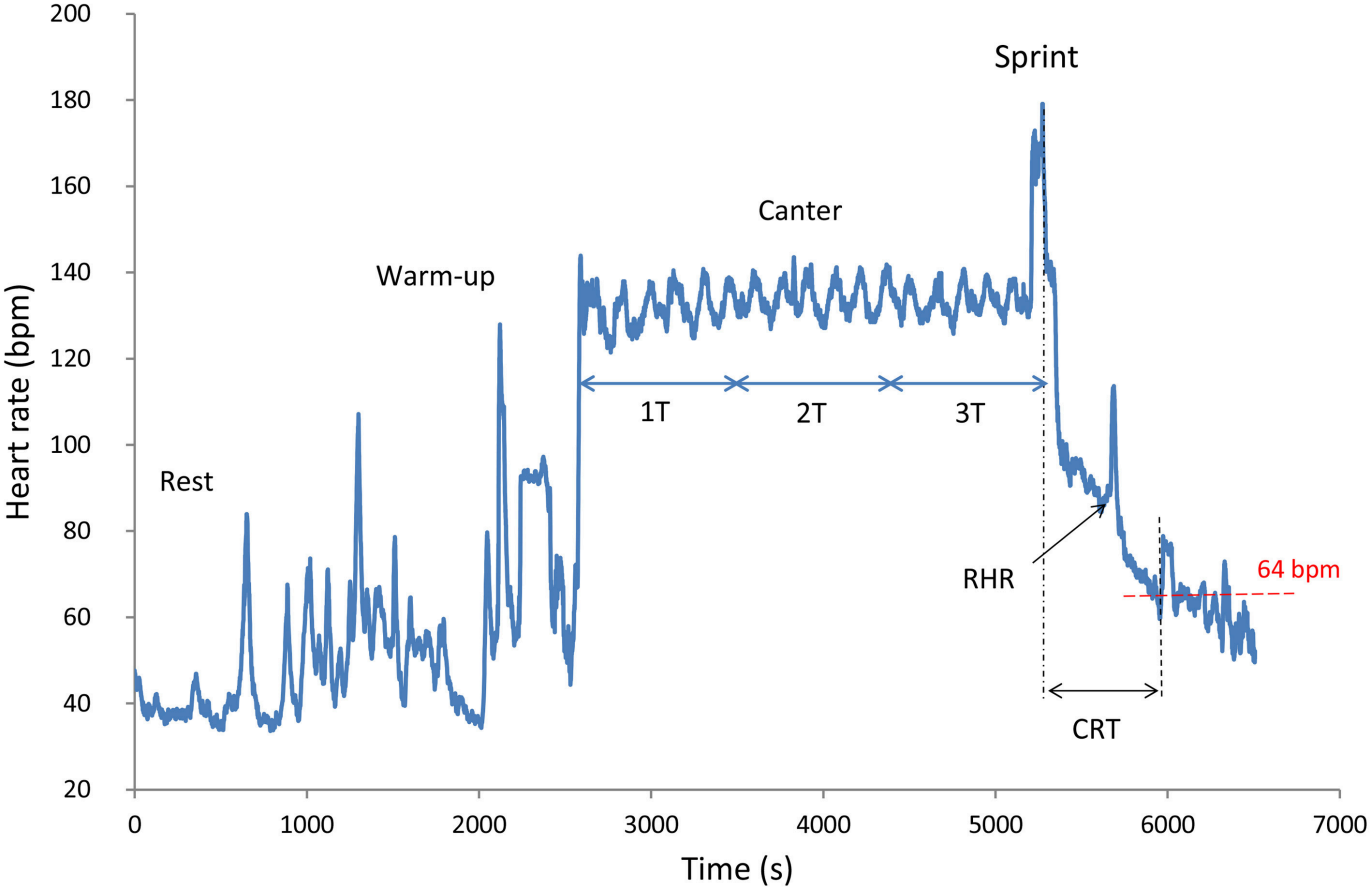
**Typical HR data recorded during a standardized field exercise test of a 6-year-old endurance horse**. *T* = 15 min (900 s). RHR, recovery heart rate; CRT, cardiac recovery time.

Exercise tests were performed in groups (composed of two or three horses of the same age) on circular training tracks with a good surface (Table 1). The exercise test began with a 15-min warm-up (10 min of walking and 5 min of trotting). The horses then performed an exercise test consisting of 15 (T), 30 (2T), or 45 min (3T) of constant-speed cantering for 4-, 5-, and 6-year-olds, respectively. The riders were asked to canter their horses at 22 km/h (~110 strides per min). The test ended with a 500 m gallop at full speed (Figure 1).

### Measurements

RR intervals were recorded at rest and during the whole exercise test with heart rate monitors (Polar S-810, Kempele, Finland). The Polar S-810 records successive RR intervals with a precision of one ms. The running speed and distance covered were recorded using the global positioning system (the Minimax GPS from Catapult Sports, Victoria, Australia).

### Signal processing

#### RR series extraction

Occasional ectopic beats (artifacts, cumulative RR periods, and extrasystoles) were identified visually and manually replaced with interpolated adjacent RR interval values. The RR recordings were then split into 15-min sequences at rest (600 R-R intervals) and during exercise (T, 2T, and 3T; T = 2000 R-R intervals) and recovery (1100 R-R intervals). Each RR sequence was analyzed using temporal and time-frequency approaches. There were not enough successive RR intervals during the final full-speed gallop to compute the smoothed pseudo-Wigner-Ville distribution (SPWVD).

#### Descriptive statistical analysis and recovery indexes

##### Temporal analysis of RR series

The mean RR interval and the standard deviation (SD) and the root-mean-square differences of successive RR intervals (RMSSD) were calculated for each 15-min sequence.

$$\text{RMSSD}=\sqrt{\frac{1}{N-1}\left[\sum_{i\;-\;1}^{N-1}{\left(RR_{i\;+\;1}-RR_{i}\right)}^{2}\right]}$$

where *N* = number of R-R interval terms.

The recovery sequences were specifically analyzed for two recovery variables: the cardiac recovery time (CRT) and the recovery heart rate (RHR). The CRT was calculated as the time difference between the end of exercise and the first instantaneous HR value lower or equal to 64 beats.min^−1^ (FEI, 2016); (Figure 1). The RHR was defined as the first instantaneous HR value recorded during the slow recovery phase (Figure 1).

#### Poincaré plot analysis

**Figure 2:**
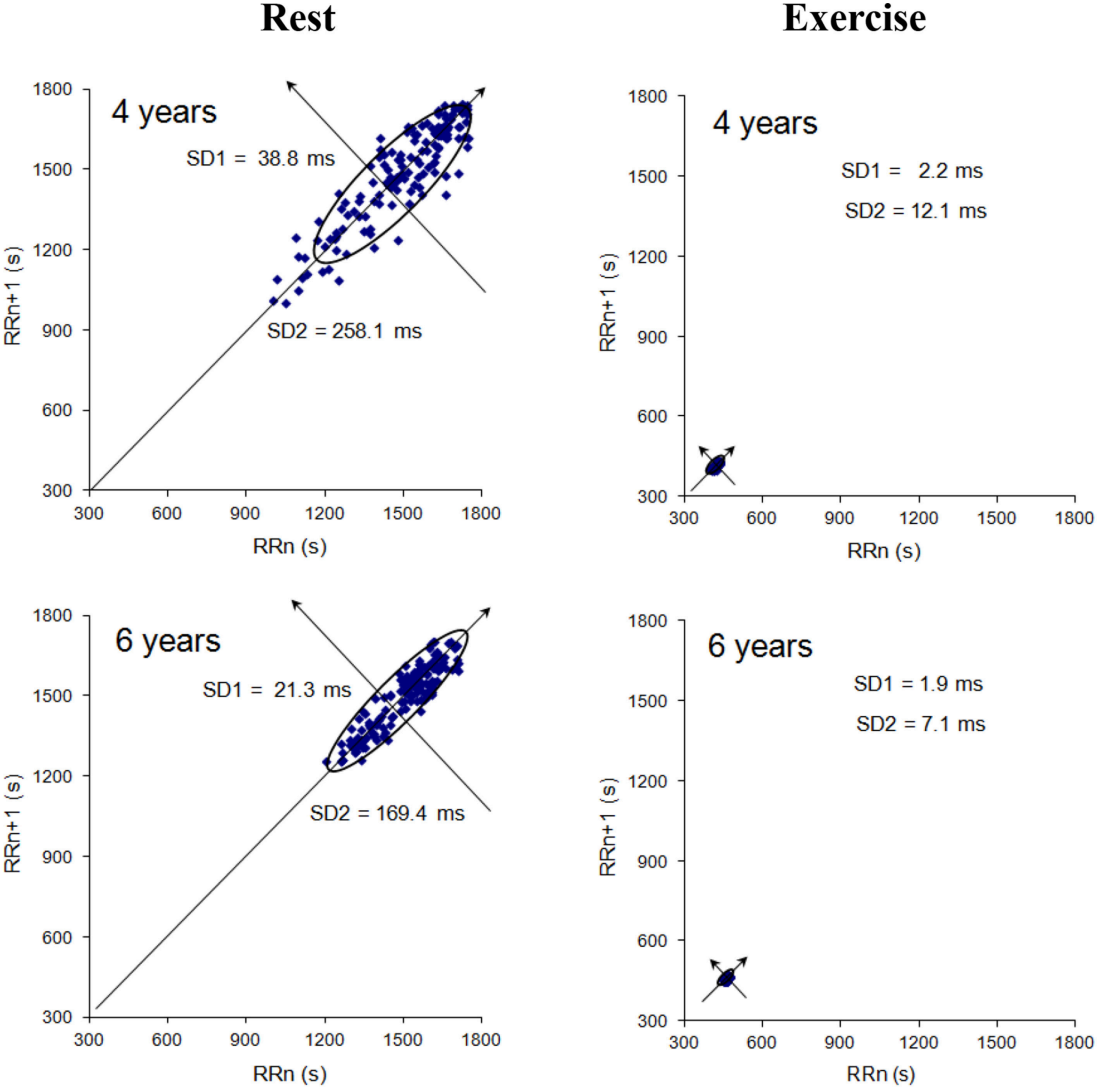
**Typical examples of Poincaré plots at rest and during exercise (for a 4-year-old in the top panel and a 6-year-old in the bottom panel)**. SD1 (the standard deviation of instantaneous variability) and SD2 (the standard deviation of continuous variability) are indicated on each plot. SD1 ($\frac{RMSSD}{\sqrt{2}}$) is lower in the 4-year-old than in the 6-year-old (*p* < 0.05).

The Poincaré plot consists in plotting each RR interval as a function of the previous RR interval (Figure 2). The shape of the resulting scatter plot makes it possible to classify HRV visually (Kamen et al., 1996). The RR interval typically appears as an elongated cloud of points oriented along the line of identity. If the scatter plot is ellipsoidal, the shape can be quantified by calculating the ellipse's width (SD1) and the length (SD2) (Cottin et al., 2004). SD1 highlights the dispersion of the points perpendicular to the line of identity and serves as an index of short-term HR variability. SD2 highlights the dispersion of the points along the line of identity and serves as an index of long-term HR variability (Brennan et al., 2001). Given that SD1 is equal to $\frac{RMSSD}{\sqrt{2}}$, it provides the same information (proportionally) as RMSSD. Hence, we did not compute SD1 *per se* in the present study.

#### SPWVD time-frequency analysis of RR series

An SPWVD time-frequency analysis was used to compute the instantaneous components of HRV. The SPWVD technique is more accurate than the short-time Fourier transform (STFT) when analysing non-stationary RR series (Monti et al., 2002).

The SPWVD analysis was performed with a cardiovascular toolbox developed in a SCILAB (Scientific Laboratory) environment (INRIA: Institut national de recherche en informatique et en automatique, France). The SPWVD provides a continuous evaluation of the amplitude and frequency and yields a near-instantaneous complex spectrum for each beat. Indeed, the SPWVD method is achieved by independent time and frequency smoothing. The SPWVD analysis used a 256-point window. The resampling rate was 4 Hz (0.25 s), and so the window's duration was 256/4 = 64 s.

In line with current standards (Camm et al., 1996; Cottin et al., 2005), the instantaneous time frequency components were computed for the low-frequency (LF: 0.04–0.15 Hz) and high-frequency (HF: 0.15–2 Hz) bands of the HRV. The spectral power was computed in LF and HF ranges (spectral components) by integrating the power spectral density (PSD), as follows:

$$LF=\sum_{f=0.04}^{0.15}PSD.\Delta f\;and\;HF=\sum_{f=0.15}^{2}PSD.\Delta f(ms^{2})$$

Furthermore, the LF and HF components were normalized, as follows:

$$\begin{array}{l} \text{LFnu}=\text{100}.\text{LF}/(\text{LF}+\text{HF})\%\text{ and}\\ \text{HFnu}=\text{100}.\text{HF}/(\text{LF}+\text{HF})\% \end{array}$$

The representation of LF and HF components in normalized units emphasizes the controlled, balanced behavior of the two branches of the autonomic nervous system (Camm et al., 1996).

### Statistics

All data were found to be normally distributed. The effects of age, exercise duration and test conditions were analyzed separately. In order to determine the effect of age on HRV variables at rest and during exercise (T: 4- vs. 5- vs. 6-year-olds; 2T: 5- vs. 6-year-olds) a one-way analysis of variance (ANOVA) was performed. The effect of test conditions on HRV variables at rest and during exercise and recovery was assessed in a one-way ANOVA. The effect of exercise duration on HRV variables was evaluated separately in 5- (T vs. 2T) and 6-year-olds (T vs. 2T vs. 3T) using a one-way repeated-measures ANOVA. Spearman's rank order correlation was also calculated. All analyses were performed with Sigma Stat software (version 3.5, 2007, Systat Software Inc. San Jose, CA, USA). The threshold for statistical significance was set to *p* < 0.05.

## Results

All horses were able to complete the exercise test without experiencing any adverse events. Only 13% of the horses participated in two or three tests, and these repetitions did not significantly influence the study results. As would be expected, the number of races increased with the horse's age: the 4-, 5-, and 6-year-olds had participated in 1.5 ± 1.5, 3.7 ± 2.2, and 7.0 ± 3.3 races, respectively. This corresponded to a total distance raced of 30 ± 29, 98 ± 63, and 277 ± 134 km, respectively. However, there was no difference between the 4-, 5-, and 6-year-olds in terms of the mean ± SD weekly duration of training (4:01 ± 2:50, 4:00 ± 1.41, and 4:41 ± 1.35 h, respectively). When analysing the HRV variables, there was no significant effect of the training level or the year of testing (Supplementary Tables 1, 2).

### Effects of age

#### At rest

The RMSSD was higher in the 4-year-olds (54.4 ± 14.5 ms) than in the 5 (44.9 ± 15.5 ms) and 6 years (49.1 ± 11.7ms) [*p* < 0.05, Degrees of freedom (DF) = 70, Table 2, Figure 2]. There were no significant intergroup differences in the other HRV variables (HR, SD2, LF, HF, LF/HF, LFnu, or HFnu; Table 2).

**Table 2:** **HRV components at rest, as a function of the age**.

	**Age (years)**		
***N* = 71**	**4**	**5**	**6**
HR (beats.mn^−1^)	45.3 ± 7.4	44.1 ± 5.2	43.8 ± 7.8
RMSSD (ms)	54.4 ± 14.5a^*^	44.9 ± 15.5 b	49.1 ± 11.7 b
SD2 (ms)	274.0 ± 88.0	222.9 ± 104.0	277.0 ± 95.0
LF (ms^2^)	499.2 ± 117.1	436.1 ± 162.9	471.5 ± 101.3
HF (ms^2^)	402.2 ± 133.9	361.1 ± 153.0	392.1 ± 101.1
LF/HF	1.3 ± 0.3	1.3 ± 0.3	1.2 ± 0.3
LFnu (%)	56.0 ± 5.6	55.1 ± 5.5	54.7 ± 5.3
HFnu (%)	44.0 ± 5.6	44.9 ± 5.5	45.3 ± 5.3

*Data are expressed as the mean ± SD*.

* *indicates a significant effect of age (p < 0.05); the mean values followed by different letters on the same line (a, b) differ significantly (p < 0.05)*.

#### During exercise

**Figure 3:**
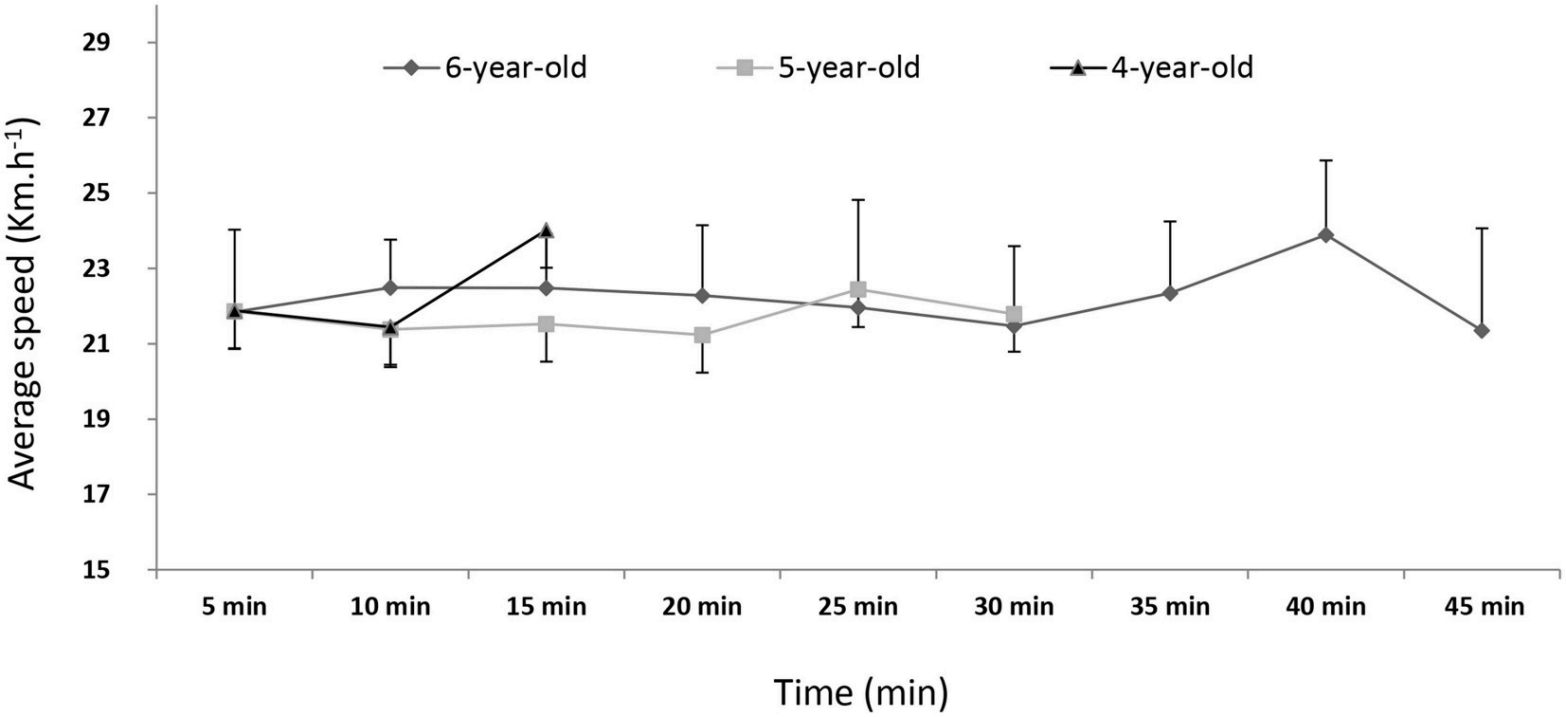
**The average cantering speed during the exercise test, as a function of age group**. Age did not have a significant effect on speed (*p* > 0.05).

There was no significant effect of age on the average cantering speed (Figure 3), which was very stable. During the first 15 min of exercise (period T), the HR and RMSSD were higher for the 4-year-olds (HR = 148.9 ± 15.0 beats.min^−1^ and RMSSD = 4.9 ± 2.8 ms) than for the 5 (HR = 142.9 ± 11.0 beats.min^−1^ and RMSSD = 3.5 ± 1.6 ms) and 6-year-olds (HR = 139.2 ± 12.7 beats.min^−1^ and RMSSD = 3.0 ± 1.4 ms), (*p* < 0.05, *DF* = 70, Table 4, Figure 2). There were no significant intergroup differences in the other HRV variables (SD2, LF, HF, LF/HF, LFnu, or HFnu). Only the 5- and 6-year-olds ran for an additional 15 min (2T). The HR was higher in the 5-year-olds than in the 6-year-olds (144.0 ± 11.2 vs. 139.6 ± 13.0 beats.min^−1^) (*p* < 0.05, *DF* = 37, Table 6) but there were no significant intergroup differences in the other HRV components (RMSSD, SD2, LF, HF, LF/HF, LFnu, or HFnu).

#### During recovery

**Figure 4:**
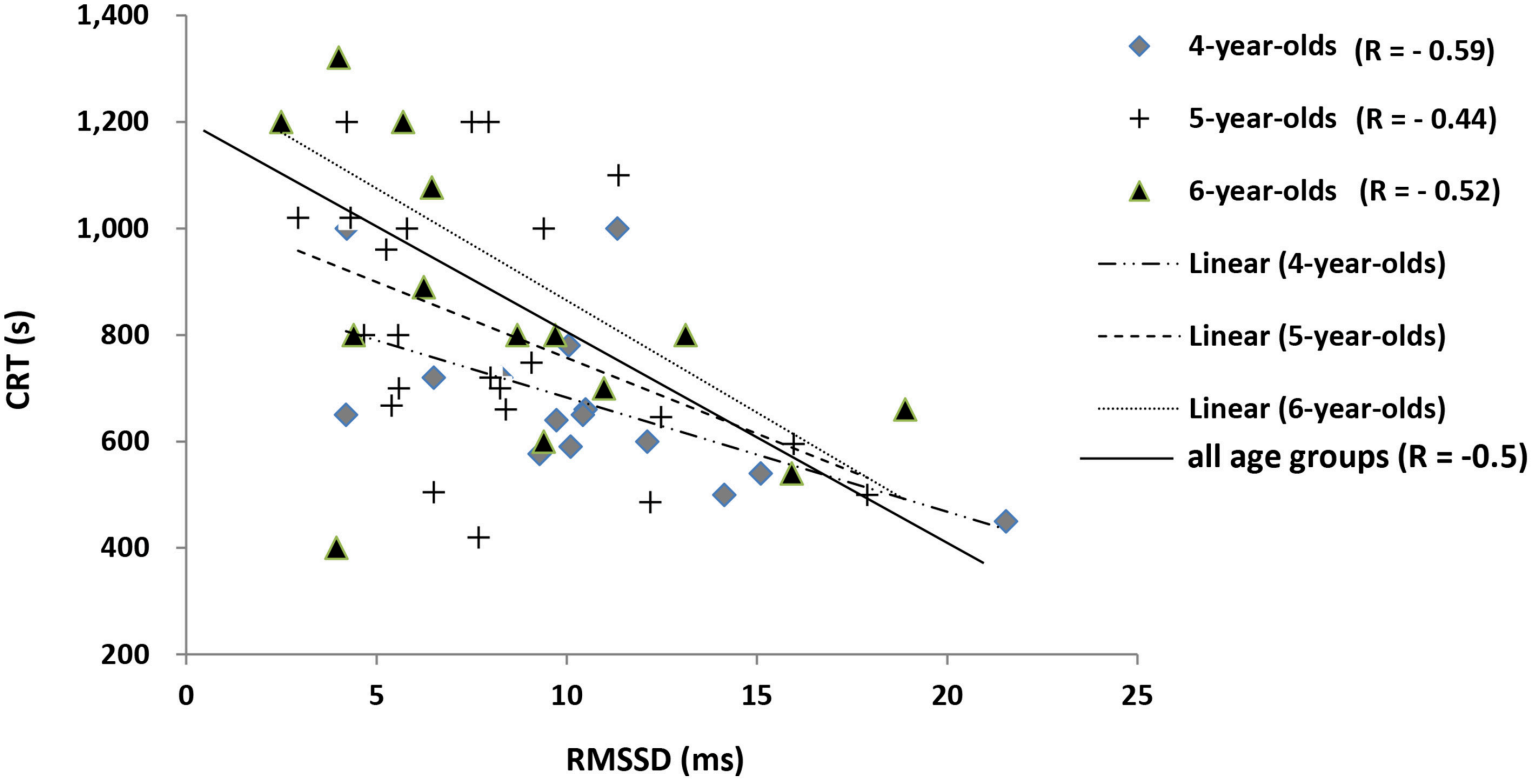
**Correlations between the mean RR, standard deviation, RMSSD, and CRT**. 4-year-old: y = −0.016x + 21.277, (*R* = −0.59; *p* < 0.05); 5-year-old: y = −0.0067x + 13.546, (*R* = −0.44; *p* < 0.05); 6-year-old: y = −0.0063x + 14.106 (*R* = −0.52; *p* < 0.05); Total (*R* = −0.5; *p* < 0.05). Horses with a higher RMSSD recovered faster.

CRT and RHR were inversely correlated with RMSSD (*R* = −0.56 and −0.53, respectively; *p* < 0.05, *n* = 61) in all age categories. This negative correlation between CRT and RMSSD was stronger in 4-year-olds (*R* = −0.59, *p* < 0.05) than in 5-year-olds (*R* = −0.44, *p* < 0.05) and 6-year-olds (*R* = −0.52, *p* < 0.05). As a result, horses with a higher RMSSD recovered more quickly and had a lower RHR (Figure 4).

### Effects of test site

#### At rest

HR was higher in A (46.9 ± 4.9 beats.min^−1^) and L (46.3 ± 5.2 beats.min^−1^) than in C2 (41.3 ± 6.6 beats.min^−1^) and C1 (40.7 ± 7.7 beats.min^−1^) (*p* < 0.05, *DF* = 70). The RMSSD was higher in C2 (59.0 ± 9.5 ms) than in A (49.5 ± 17.3 ms), L (42.8 ± 10.9 ms), and C1 (50.1 ± 14.8 ms) (*p* < 0.05, *DF* = 70, Table 3). Furthermore, the ratio LF/HF and LFnu (%) were higher at site A (LF/HF: 1.4 ± 0.4; LFnu: 57.3 ± 6.1) than at sites C2 (LF/HF: 1.3 ± 0.2; LFnu: 56.1 ± 3.2), L (LF/HF: 1.3 ± 0.2; LFnu: 55.4 ± 4.8), and C1 (LF/HF: 1.1 ± 0.2; LFnu: 52.1 ± 4.9) (*p* < 0.05, *DF* = 70, Table 3). However, the HFnu was higher in C1 (47.9 ± 4.9) than in A (42.7 ± 6.1), C2 (43.9 ± 3.2), and L (44.6 ± 4.8) (*p* < 0.05, *DF* = 70, Table 3)

**Table 3:** **HRV components at rest, as a function of the test site**.

***N* = 71**	**Test site**			
	**A**	**C2**	**L**	**C1**
HR (beats.mn^−1^)	46.9 ± 4.9a^*^	41.3 ± 6.6b	46.3 ± 5.2a	40.7 ± 7.7b
RMSSD (ms)	49.5 ± 17.3b	59.0 ± 9.5a^*^	42.8 ± 10.9b	50.1 ± 14.8b
SD2 (ms)	260.8 ± 115.0	290.9 ± 120.0	221.3 ± 83.2	211.2 ± 78.0
LF (ms^2^)	495.9 ± 145.3	472.1 ± 96.7	447.2 ± 143.7	426.3 ± 144.4
HF (ms^2^)	380.6 ± 152.3	371.4 ± 89.1	367.4 ± 139.8	393.3 ± 137.9
LF/HF	1.4 ± 0.4a^*^	1.3 ± 0.2b	1.3 ± 0.2b	1.1 ± 0.2b
LFnu (%)	57.3 ± 6.1a^*^	56.1 ± 3.2b	55.4 ± 4.8b	52.1 ± 4.9b
HFnu (%)	42.7 ± 6.1b	43.9 ± 3.2b	44.6 ± 4.8b	47.9 ± 4.9a^*^

*Data are expressed as the mean ± SD*.

* *indicates a significant effect of the test site (p < 0.05); the mean values followed by different letters on the same line (a, b) differ significantly (p < 0.05)*.

**Table 4:** **HRV variables during T (15 min), according to age**.

***N* = 54**	**Age (years old)**		
	**4**	**5**	**6**
Average speed (km.h^−1^)	22.2 ± 1.0	21.8 ± 1.6	22.4 ± 0.8
HR (beats.mn^−1^)	148.9 ± 15.0a^*^	142.9 ± 11.0b	139.2 ± 12.7b
RMSSD (ms)	4.9 ± 2.8a^*^	3.5 ± 1.6b	3.0 ± 1.4b
SD2 (ms)	28.8 ± 18.7	27.16 ± 18.6	35.4 ± 17.14
LF (ms^2^)	11.2 ± 3.9	11.2 ± 6.2	8.7 ± 2.36
HF (ms^2^)	11.2 ± 8.3	11.4 ± 6.1	8.4 ± 4.5
LF/HF	1.4 ± 0.7	1.1 ± 0.5	1.3 ± 0.7
LFnu (%)	54.8 ± 14.6	50.4 ± 15.2	52.6 ± 13.6
HFnu (%)	45.2 ± 14.6	49.6 ± 15.2	47.4 ± 13.6

*Data are expressed as the mean ± SD*.

* *indicates a significant effect of age (p < 0.05); the mean values followed by different letters on the same line (a, b) differ significantly (p < 0.05)*.

#### During exercise

During the first 15 min of exercise (T), the mean running speed and RMSSD varied according to the test site (*p* < 0.05, *DF* = 53, Table 5). The running speed was higher at sites A (23.1 ± 1.3 km.h^−1^) and C1 (22.4 ± 0.9 km.h^−1^) than at sites C2 (21.1 ± 1.0 km.h^−1^) and L (21.2 ± 0.8 km.h^−1^) (*p* < 0.05, *DF* = 53, Table 5). The RMSSD was higher at site A (6.4 ± 2.7 ms) than at sites C2 (3.2 ± 1.0 ms), L (2.7 ± 1.3 ms), and C1 (4.0 ± 1.6 ms) (*p* < 0.05, *DF* = 53, Table 5). There were no differences between the test sites in terms of the other HRV variables (HR, SD2, LF, HF, LF/HF, LFnu, and HFnu, Table 5). For the 5- and 6-year-olds, the mean running speed (AS) and HR during the additional 15-min bout (2T) were higher at site C1 (AS: 23.1 ± 1.7 km.h^−1^; HR: 148.5 ± 10.7 beats.min^−1^) than at sites C2 (AS: 21.1 ± 0.6 km.h^−1^; HR: 137.5 ± 6.0 beats.min^−1^) and L (AS: 20.7 ± 1.0 km.h^−1^; HR: 138.1 ± 11.5 beats.min^−1^) (*p* < 0.05, *DF* = 37, Table 7). However, the LF power of HRV was higher at site C2 (13.8 ± 6.9 ms^2^) than at sites L (7.7 ± 2.3 ms^2^) and C1 (10.9 ± 5.3 ms^2^) (*p* < 0.05, *DF* = 37, Table 7). There were no significant differences between the test sites in terms of the other HRV variables (RMSSD, SD2, HF, LF/HF, LFnu, and HFnu, Table 7).

**Table 5:** **HRV variables during T (15 min) according to the test site**.

***N* = 54**	**Test site**			
	**A**	**C2**	**L**	**C1**
Average speed (km.h^−1^)	23.1 ± 1.3a^*^	21.1 ± 1.0b	21.2 ± 0.8b	22.4 ± 0.9a
HR (beats.mn^−1^)	142.2 ± 15.2	140.0 ± 9.3	141.2 ± 13.7	146.9 ± 1
RMSSD (ms)	6.4 ± 2.7a^*^	3.2 ± 1.0b	2.7 ± 1.3c	4.0 ± 1.6b
SD2 (ms)	38.3 ± 12.1	34.2 ± 23.2	23.7 ± 17.8	34.8 ± 16.7
LF (ms^2^)	12.6 ± 3.3	11.2 ± 3.9	6.9 ± 1.5	13.7 ± 5.8
HF (ms^2^)	9.3 ± 6.5	8.1 ± 5.1	8.7 ± 5.5	13.8 ± 6.6
LF/HF	1.6 ± 0.7	1.6 ± 0.6	1.1 ± 0.6	1.2 ± 0.6
LFnu (%)	60.1 ± 11.8	60.3 ± 8.9	48.4 ± 15.7	51.0 ± 14.4
HFnu (%)	39.9 ± 11.8	39.7 ± 8.9	51.6 ± 15.7	49.0 ± 14.4

*Data are expressed as the mean ± SD*.

* *indicates a significant effect of test site (p < 0.05); the mean values followed by different letters on the same line (a, b, c) differ significantly (p < 0.05)*.

**Table 6:** **HRV variables during 2T (2 × 15 min), as a function of the age**.

***N* = 38**	**Age (years)**	
	**5**	**6**
Average speed (km.h^−1^)	22.1 ± 1.8	22.2 ± 1.6
HR (beats.mn^−1^)	144.0 ± 11.2a^*^	139.6 ± 13.0b
RMSSD (ms)	3.1 ± 1.5	3.6 ± 2.2
SD2 (ms)	21.4 ± 10.1	19.8 ± 6.1
LF (ms^2^)	9.6 ± 4.6	10.0 ± 5.2
HF (ms^2^)	10.4 ± 6.6	8.4 ± 4.6
LF/HF	1.2 ± 0.7	1.4 ± 0.8
LFnu (%)	49.8 ± 16.3	54.7 ± 14.2
HFnu (%)	50.2 ± 16.3	45.3 ± 14.2

*Data are expressed as the mean ± SD*.

* *indicates a significant effect of age or test site (p < 0.05); the mean values followed by different letters on the same line (a, b) differ significantly (p < 0.05)*.

**Table 7:** **HRV variables during 2T (2 × 15 min), as a function of the test site**.

***N* = 38**	**Test site**		
	**C2**	**L**	**C1**
Average speed (km.h^−1^)	21.1 ± 0.6b	20.7 ± 1.0b	23.1 ± 1.7a^*^
HR (beats.mn^−1^)	137.5 ± 6.0b	138.1 ± 11.5b	148.5 ± 10.7a^*^
RMSSD (ms)	4.7 ± 3.5	2.5 ± 1.0	3.7 ± 1.7
SD2 (ms)	20.7 ± 4.4	15.0 ± 3.7	24.8 ± 8.7
LF (ms^2^)	13.8 ± 6.9a^*^	7.7 ± 2.3b	10.9 ± 5.3b
HF (ms^2^)	6.3 ± 2.1	8.1 ± 4.3	13.5 ± 7.5
LF/HF	2.1 ± 0.7	1.2 ± 0.7	1.1 ± 0.6
LFnu (%)	66.8 ± 8.3	50.8 ± 15.3	46.2 ± 15.0
HFnu (%)	33.2 ± 8.3	49.2 ± 15.3	53.8 ± 15.0

*Data are expressed as the mean ± SD*.

* *indicates a significant effect of test site (p < 0.05); the mean values followed by different letters on the same line (a, b) differ significantly (p <0.05)*.

#### During recovery

CRT was lower at sites C2 (631.5 ± 90.3 s) and A (625.6 ± 214.0 s) than at sites L (904.0 ± 237.0 s) and C1 (815.5 ± 354.0 s) (*p* < 0.05, *DF* = 60, Table 8). The recovery RMSSD was higher at C2 (16.1 ± 13.5 ms) than at A (13.1 ± 4.9 ms), L (7.9 ± 4.1 ms), and C1 (7.5 ± 3.6 ms) (*p* < 0.05, *DF* = 60, Table 8). The LF power during recovery was higher at A (258.9 ± 145.5 ms^2^) than at C2 (265.0 ± 65.7 ms^2^), L (177.3 ± 61.7 ms^2^), and C1 (216.0 ± 76.9 ms^2^) (*p* < 0.05, *DF* = 60, Table 8). There were no significant differences between the test sites in terms of the other HRV variables (SD2, HF, LF/HF, LFnu, and HFnu).

**Table 8:** **HRV variables during cardiac recovery, as a function of the test site**.

***N* = 61**	**Test site**			
	**A**	**C2**	**L**	**C1**
RHR (beats.mn^−1^)	91.5 ± 15.1	85.2 ± 11.0	99.7 ± 15.0	100.3 ± 13.6
CRT (s)	624.6 ± 214.0b	631.5 ± 90.3b	904.0 ± 237.0a^*^	815.5 ± 354.0a
RMSSD (ms)	13.1 ± 4.9b	16.1 ± 13.5a^*^	7.9 ± 4.1c	7.5 ± 3.6c
SD2 (ms)	196.8 ± 82.6	163.1 ± 102.2	173.0 ± 79.0	140.7 ± 59.1
LF (ms^2^)	258.9 ± 145.5a^*^	265.0 ± 65.7b	177.3 ± 61.7b	216.0 ± 76.9b
HF (ms^2^)	135.2 ± 83.2	152.3 ± 40.2	108.9 ± 60.9	127.0 ± 38.8
LF/HF	2.2 ± 1.0	1.8 ± 0.4	1.9 ± 0.7	1.7 ± 0.4
LFnu (%)	66.3 ± 10.5	63.4 ± 5.4	63.3 ± 8.4	62.4 ± 5.2
HFnu (%)	33.7 ± 10.5	36.6 ± 5.4	36.7 ± 8.4	37.6 ± 5.2

*Data are expressed as the mean ± SD*.

* *indicates a significant effect of test site (p < 0.05); the mean values followed by different letters on the same line (a, b, c) differ significantly (p < 0.05)*.

### Effects of the exercise duration

The 5- and 6-year-olds cantered for at least 30 min (2T) and only the 6-year-olds cantered for 45 min (3T). Therefore, the effect of the exercise duration on HRV components could not be tested in the 4-year-olds. In the 5-year-olds, we did not observe a significant effect of exercise duration on the average cantering speed or any of the HRV components when comparing T and 2T durations (Table 9). In the 6-year-olds, RMSSD was lower in 3T (2.8 ± 1.0 ms) than in 2T (3.6 ± 2.2) and T (3.0 ± 1.4) (*P* < 0.05, *DF* = 17, Table 9), with no difference between 2T and T. SD2 was higher for T (35.4 ± 17.1 ms) than for 2T (19.8 ± 6.1) and 3T (29.7 ± 10) (*p* < 0.05, *DF* = 17, Table 9). There were no differences in the other HRV components (HR, LF, HF, LF/HF, LFnu, and HFnu) when comparing T, 2T, and 3T in the 6-year-old group (Table 9).

**Table 9:** **Effect of exercise duration on HRV variables**.

***N* = 38**	**5 years old**		**6 years old**		
	**T**	**2T**	**T**	**2T**	**3T**
Average speed (km.h^−1^)	21.8 ± 1.6	22.1 ± 1.8	22.4 ± 0.8	22.2 ± 1.6	22.2 ± 1.9
HR (beats.mn^−1^)	142.9 ± 11.0	143.9 ± 11.2	139.2 ± 12.0	139.6 ± 13.0	144.9 ± 15.0
RMSSD (ms)	3.5 ± 1.6	3.1 ± 1.5	3.0 ± 1.4b	3.6 ± 2.2b	2.8 ± 1.0a^*^
SD2 (ms)	27.1 ± 18.6	21.4 ± 10.1	35.4 ± 17.1a^*^	19.8 ± 6.1b	29.7 ± 10.1b
LF (ms^2^)	11.2 ± 25.0	9.6 ± 4.6	8.7 ± 2.3	10.0 ± 5.2	9.9 ± 4.6
HF (ms^2^)	11.4 ± 6.1	10.4 ± 6.6	8.4 ± 4.5	8.4 ± 4.6	8.1 ± 4.2
LF/HF	1.2 ± 0.5	1.2 ± 0.7	1.3 ± 0.7	1.4 ± 0.8	1.4 ± 0.6
LFnu (%)	50.4 ± 15.2	49.8 ± 16.3	52.6 ± 13.6	54.7 ± 14.2	55.7 ± 11.3
HFnu (%)	49.6 ± 15.2	50.2 ± 16.3	47.4 ± 13.6	45.3 ± 14.2	44.3 ± 11.3

*Data are expressed as the mean ± SD*.

* indicates a significant effect of age or test site

*(p < 0.05); the mean values followed by different letters on the same line (a, b) differ significantly (p < 0.05)*.

## Discussion

By analyzing a large dataset of HR recordings (in 77 young endurance horses performing exercise tests at four different sites), we generated new information on the effects of age, test conditions, and exercise duration on HRV in endurance horses. Our main finding was that HRV amplitude decreased as the age and exercise duration increased. Furthermore, HRV analysis during recovery might be of value for selecting the fittest young endurance horses (i.e., those with the fastest cardiac recovery within 20 min of arrival at a vet gate).

Blocks of 15-min RR recordings at rest and during exercise and recovery were analyzed with methods for quantifying HRV in a nonlinear analysis (the Poincaré plot) and in the time-frequency domain (computation of the SPWVD). The major advantages of the Poincaré plot are its ability to provide an overall preview of HRV and its relative insensitivity to the RR interval recording quality (Kamen et al., 1996). This method has been used to study the effect of age and physical fitness on HRV components in humans (Tulppo et al., 1998) and to test the effect of exercise repetitions on recovery in the horse (Cottin et al., 2006). The SPWVD technique is more accurate than STFT for time-frequency analysis. STFT is affected by the Heisenberg-Gabor uncertainty principle, which states that when the accuracy in the time domain is high, the accuracy in the frequency domain is low (and vice versa). In contrast, SPWVD has identical levels of accuracy in both the time and frequency domains—making it more able to cope with non-stationarity observed in this study during exercise and recovery period. Hence, this type of time-frequency method is very suitable for analyzing time series during exercise (Cottin et al., 2008; Filliau et al., 2014).

There are few published data on the effect of a horse's age on HRV. At rest, some studies have shown that the HR decreases and the RMSSD increases between the ages of 9 and 22 months (Visser et al., 2002). The results of the present study show that in older horses (aged 4–6), HR at rest did not significantly vary with age, whereas RMSSD decreased with increasing age. The discrepancy between Visser et al.'s results and our findings may be attributed to inter-study differences in age and breed (Dutch Warmbloods under the age of 2 and 4- to 6-year-old Arabian horses, respectively). In humans, it is well known that HR and the amplitude of HRV (particularly the HF modulation of HRV, related to respiratory sinus arrhythmia) decrease between adolescence and old age (Brandenberger et al., 2003; Filliau et al., 2014). The lack of such changes in the present study of horses is probably due to the smaller relative age difference and the more similar levels of physical fitness. However, we did observe a lower RMSSD in 6-year-olds than in 4-year-olds; this may correspond to the start of an age-related decrease in HRV similar to that observed in humans (Silvetti et al., 2001; Brandenberger et al., 2003; Filliau et al., 2014).

During exercise, we found that the HR was highest in the youngest horses. Firstly, the HR was higher in 4-year-olds than in 5- and 6-year-olds during the first 15 min of cantering; secondly, the HR was higher in 5-year-olds than in 6-year-olds during a 30-min canter. In a study of endurance horses, Trachsel et al. (2014) showed that heart size and stroke volume increased between the ages of 4 and 6. As the cantering speed during the exercise test was steady (22 km.h^−1^, Figure 3), for a given cardiac output, a higher stroke volume may explain a lower HR in the older horses. However, a study in Standardbred horses (Betros et al., 2002) reported that HR_max_, VO_2_max and the velocities at HR_max_ and VO_2_max were similar in young and middle-aged horses but were lower in old horses. Furthermore, a study of event horses showed that individuals with a lower HR during a standardized exercise test were ranked more highly in competitions (Auvinet et al., 1991).

The effects of exercise duration were investigated in the 5-year-olds (T vs. 2T) and in the 6-year-olds (T vs. 2T vs. 3T). We found that both SD2 and RMSSD decreased with exercise duration. SD2 is proportional to the long-term variability in an RR series (Brennan et al., 2001), and RMSSD reflects the modulation of HR by breathing. In humans, SD2 and RMSSD (SD1) were markedly lower during a progressive vagal blockade by atropine infusion (Tulppo et al., 1996). During complete vagal blockade, both SD2 and RMSSD fell as the exercise intensity increased (up until VO_2_max) (Tulppo et al., 1996). Furthermore, it has been shown (Hautala et al., 2001) that both RMSSD and SD2 are significantly lower during the first night after a 75 km cross-country skiing race than during the night before the race. It has therefore been suggested that RMSSD and SD2 are valuable markers of fatigue and vagal withdrawal during exercise in humans (Hautala et al., 2001). In horses, it has been demonstrated that RMSSD decreased during intermittent, high-speed trotting, whereas SD2 remained stable (Cottin et al., 2006). However, SD2 decreased during the repeated recoveries (Cottin et al., 2006). Our present findings confirm these results; low RMSSD and SD2 values may be reliable markers of fatigue and poor recovery in endurance horses.

During recovery period, the RHR and CRT are reliable indexes of the training level and cardiac health in horses and humans (Tomlin and Wenger, 2001; Borresen and Lambert, 2008). During acute exercise, recovery variables may provide useful markers of fatigue in horses (Cottin et al., 2006; Hada et al., 2006; Bitschnau et al., 2010). In a study of endurance horses, Madsen et al. (2014) reported that the CRT (i) was lowest in the most trained individuals and (ii) increased with the exercise intensity. In the present study, it was difficult to draw solid conclusions concerning post-exercise cardiac recovery as a function of age because the exercise duration differed from one age group to another. However, horses with the lowest recovery HR and CRT values had the highest recovery RMSSD. Accordingly, RMSSD (a vagal component) may be a reliable, useful index of post-exercise cardiac recovery in endurance horses.

As expected, the test location influenced the HRV components and the speed. The weather conditions (particularly the ambient temperature and track quality) might have had an effect on the HRV components. It has been shown (McConaghy et al., 1996) that ponies were able to maintain body temperature in a hot environment by increasing blood flow to the tissues involved in heat dissipation, whereas blood flow to all other tissues remained stable. This was achieved by increasing the cardiac output. Thus, an increase in the ambient temperature and humidity might induce an increase in HR. Although the ambient temperature was highest and horses displayed the highest resting HR at test site A (27°C), the horses at site L displayed a similar resting HR at a lower ambient temperature (18°C) and a higher humidity level (73%, vs. 40% at site A). It has been shown that horses in a hot, humid environment breathe more rapidly than in cold conditions (Marlin et al., 2001). Furthermore, hot, humid conditions increase the risk of metabolic failure during endurance competitions via electrolyte depletion and disruption of thermoregulatory mechanisms (Robert et al., 2010). Several studies have found that weather conditions have a direct impact on performance in endurance competitions (Marlin et al., 1995; McCutcheon and Geor, 2010; Nagy et al., 2014). Accordingly, our present results confirmed that weather conditions impact HRV in endurance horses and may influence performance. However, differences in weather conditions cannot fully explain the observed difference in HRV components. The running speed was higher at sites A and C1 than at sites C2 and L. Other research has shown that the ground surface has an effect on stride parameters (Setterbo et al., 2009). A difference in the type of track surface (hard at sites A and C2 and soft at sites C1 and L) may account for some of the observed differences between test sites. A further explanatory factor may be the horses' age distribution; the 4-year old horses were less well trained, making it more difficult to control their speed. This may explain why the speed was higher at site A (where the horses were younger). It is noteworthy that the highest HRV (RMSSD) value was reported at site A (i.e., with younger horses and the highest mean cantering speed).

The duration of the field exercise test was chosen as a function of the horse's age, category and physical ability, in order to avoid accidents and decrease the likelihood of post-test metabolic disorders. However, the field exercise test has a number of limitations relative to laboratory-based exercise conditions (such as treadmill tests) because it was neither possible to fully control the cantering speed or the environmental conditions. Although, the average cantering speed varied slightly according to sites, there was no significant difference in average speed between the three age groups. An increase in speed is associated with an increased heart rate and thus a decrease in the various HRV components. To better standardize speeds, riders could use GPS to adjust the speed of their horses during the tests. To minimize the environmental effects, the exercise tests should also be performed on the same track or on tracks with similar characteristics (length, type of soil), and in the same climatic conditions.

## Conclusion

In the present study of 4- to 6-year-old endurance horses, we found that the HR and HRV (RMSSD) at rest and during exercise were higher in 4-year-olds than in 5- or 6-year-olds. Furthermore, the HRV decreased as the exercise duration increased. However, the measured HRV components appear to be strongly influenced by the environmental recording conditions (such as ambient temperature, humidity and the type of track surface). As observed in humans, the RMSSD component may be a reliable, useful index of fatigue and recovery in endurance horses. Accordingly, HR and RMSSD may be of value in selecting the best young endurance horses (i.e., those with the fastest cardiac recovery during a standardized exercise test). These results should be confirmed on a larger number of subjects. Moreover, they can be correlated with performance in endurance competition in adulthood to determine their predictive value of the quality of horses. Thus, HRV parameters could be used to establish the relative abilities of horses of the same age examined the same day on the same place.

## Author contributions

MY prepared the database, performed the statistical analysis, and wrote the manuscript. CR was involved in designing the study and collecting the data. EB conceived and designed the study, performed the statistical analysis. FC helped to analyze the HR data, draft the manuscript, and wrote the manuscript. The final manuscript was read and approved by all the co-authors.

## Funding

This study was part of the GenEndurance project, which was financially supported by the “FondsEperon,” the “InstitutFran7ais du Cheval et de l'Equitation” (IFCE), “the Association du Cheval Arabe” (ACA), the “Institut Nationalde Recherche Agronomique” (INRA) and the “EcoleNationaleV9t9rinaired'Alfort” (ENVA).

### Conflict of interest statement

The authors declare that the research was conducted in the absence of any commercial or financial relationships that could be construed as a potential conflict of interest.
