# Supplementary material for: Effects of Age, Exercise Duration, and Test Conditions on Heart Rate Variability in Young Endurance Horses
Source: Front Physiol. 2016 May 2;7:155. doi: 10.3389/fphys.2016.00155 (PMC4852288; doi:10.3389/fphys.2016.00155)
Supplement: Supplementary file 1 [file Table1.DOC]

Table 1. HRV components as a function of the weekly duration of training

|  |  | weekly duration of training | | |
| --- | --- | --- | --- | --- |
|  | N = 77 | 0- 2 hours | 3- 4 hours | 5-7 hours |
| Rest | HR (beatsmn^-1^) | 42.9±6.3 | 44.5±7.1 | 46.8±5.9 |
|  | RMSSD (ms) | 52.9±14.6 | 46.8±12.0 | 43.7±16.3 |
|  | SD2 (ms) | 255.5±95.0 | 223.3±106.6 | 235.3±104.9 |
|  | LF (ms²) | 468.6±107.7 | 405.6±128.0 | 498.3±177.5 |
|  | HF (ms²) | 384.8±136.5 | 350.8±99.7 | 396.0±164.1 |
|  | LF/HF | 1.2±0.2 | 1.1±0.2 | 1.3±0.3 |
|  | LFnu (%) | 55.6±5.0 | 53.4±5.6 | 56.2±5.7 |
|  | HFnu (%) | 44.4±5.0 | 46.6±5.6 | 43.8±5.7 |
| Exercise | HR (beatsmn^-1^) | 141.7±11.2 | 145.3±12.9 | 142.8±17.6 |
|  | RMSSD (ms) | 3.0±1.3 | 3.5±1.6 | 3.7±1.6 |
|  | SD2 (ms) | 25.0±14.6 | 27.4±19.6 | 37.7±16.5 |
|  | LF (ms²) | 9.7±3.3 | 11.1±7.2 | 13.5±5.7 |
|  | HF (ms²) | 7.9±4.8 | 15.1±6.0 | 12.8±7.3 |
|  | LF/HF | 1.4±0.6 | 0.8±0.6 | 1.1±0.4 |
|  | LFnu (%) | 57.3±10.5 | 40.9±17.1 | 52.6±7.5 |
|  | HFnu (%) | 42.7±10.5 | 59.1±17.1 | 47.4±7.5 |
| Recovery | HR (beatsmn^-1^) | 95.0±12.8 | 99.5±16.3 | 96.0±15.5 |
|  | RMSSD (ms) | 10.8±7.8 | 8.2±3.5 | 10.2±5.6 |
|  | SD2 (ms) | 146.4±80.2 | 173.7±67.8 | 195.9±88.1 |
|  | LF (ms²) | 213.6±84.8 | 210.0±72.3 | 227.9±143.0 |
|  | HF (ms²) | 114.9±52.9 | 130.7±58.5 | 137.3±74.2 |
|  | LF/HF | 2.0±0.6 | 1.7±0.5 | 1.8±0.9 |
|  | LFnu (%) | 65.9±6.6 | 61.9±6.9 | 61.5±10.0 |
|  | HFnu (%) | 34.1±6.6 | 38.1±6.9 | 38.5±10.0 |

Data are expressed as the mean ± SD
