# Supplementary material for: Effects of Age, Exercise Duration, and Test Conditions on Heart Rate Variability in Young Endurance Horses
Source: Front Physiol. 2016 May 2;7:155. doi: 10.3389/fphys.2016.00155 (PMC4852288; doi:10.3389/fphys.2016.00155)
Supplement: Supplementary file 2 [file Table2.DOC]

Table 2. HRV components as a function of the year of testing.

|  |  | Year of testing | | |
| --- | --- | --- | --- | --- |
|  | N = 77 | 2012 | 2013 | 2014 |
| Rest | HR (beats.mn^-1^) | 45.6±5.1 | 45.5±6.7 | 41.5±7.2 |
|  | RMSSD (ms) | 44.6**±**12.5 | 50.2±15.3 | 51.2±15.8 |
|  | SD2 (ms) | 232.8±99.7 | 243.1±107.3 | 243.7±94.4 |
|  | LF (ms²) | 466.2±168.0 | 460.3±134.5 | 454.8±114.9 |
|  | HF (ms²) | 378.9±139.2 | 376.6±135.9 | 382.7±140.8 |
|  | LF/HF | 1.2±0.2 | 1.3±0.3 | 1.2±0.2 |
|  | LFnu (%) | 55.1±5.0 | 55.5±6.5 | 54.9±4.4 |
|  | HFnu (%) | 44.9±5.0 | 44.5±6.5 | 45.1±4.4 |
| Exercise | HR (beats.mn^-1^) | 148.6±14.9 | 140.4±13.6 | 140.4±9.1 |
|  | RMSSD (ms) | 4.8±1.3 | 3.0±1.6 | 2.6±0.8 |
|  | SD2 (ms) | 29.0±11.7 | 32.7±21.9 | 24.5±15.2 |
|  | LF (ms²) | 13.6±7.9 | 10.1±4.3 | 9.5±3.4 |
|  | HF (ms²) | 15.6±3.9 | 12.3±7.7 | 7.7±4.7 |
|  | LF/HF | 0.9±0.7 | 1.0±0.5 | 1.4±0.6 |
|  | LFnu (%) | 43.6±19.8 | 47.7±12.5 | 57.2±10.6 |
|  | HFnu (%) | 56.4±19.8 | 52.3±12.5 | 42.8±10.6 |
| Recovery | HR (beats.mn^-1^) | 98.1±15.8 | 98.4±16.6 | 92.6±10.9 |
|  | RMSSD (ms) | 10.3±4.5 | 8.4±4.3 | 11.1±9.0 |
|  | SD2 (ms) | 168.9±66.7 | 187.0±83.3 | 137.2±80.8 |
|  | LF (ms²) | 232.0±115.0 | 213.8±97.0 | 207.4±79.7 |
|  | HF (ms²) | 134.8±48.0 | 127.6±74.6 | 11.7±48.5 |
|  | LF/HF | 1.8±0.8 | 1.8±0.7 | 1.9±0.5 |
|  | LFnu (%) | 62.4±9.1 | 63.4**±**7.8 | 65.3±6.3 |
|  | HFnu (%) | 37.6±9.1 | 36.5±7.8 | 34.7±6.3 |

Data are expressed as the mean ± SD.
